# Impact of multimodal strategies including a pay for performance strategy in the improvement of infection prevention and control practices in healthcare facilities during an Ebola virus disease outbreak

**DOI:** 10.1186/s12879-022-07956-5

**Published:** 2023-01-06

**Authors:** Landry Kabego, Mamadou Kourouma, Kevin Ousman, April Baller, Jean-Paul Milambo, John Kombe, Bienvenu Houndjo, Franck Eric Boni, Castro Musafiri, Siya Molembo, Stéphanie Kalumuna, Moise Tshongo, John Ndizihiwe Biringiro, Nancy Moke, Clarisse Kumutima, Justin Nkita, Polydor Ngoma, Chedly Azzouz, Raphaël Okum, Michel Yao, Dick Chamla, Abdou Salam Gueye, Ibrahima Soce Fall

**Affiliations:** 1 World Health Organization, Kinshasa, Democratic Republic of the Congo; 2 Ministry of Health, Kinshasa, Democratic Republic of the Congo; 3 United Nations International Children’s Funds, Kinshasa, Democratic Republic of the Congo; 4 Africa Centre for Disease Control, Kinshasa, Democratic Republic of the Congo

**Keywords:** Pay per performance, Infection prevention and control, Healthcare worker infections

## Abstract

**Background:**

Strategy to mitigate various Ebola virus disease (EVD) outbreaks are focusing on Infection Prevention and Control (IPC) capacity building, supportive supervision and IPC supply donation. This study was conducted to assess the impact of a Pay for Performance Strategy (PPS) in improving IPC performance in healthcare facilities (HF) in context of the 2018–2019 Nord Kivu/ Democratic Republic of the Congo EVD outbreak.

**Methods:**

A quasi-experimental study was conducted analysing the impact of a PPS on the IPC performance. HF were selected following the inclusion criteria upon informed consent from the facility manager and the National Department of Health. Initial and process assessment of IPC performance was conducted by integrating response teams using a validated IPC assessment tool for HF. A bundle of interventions was then implemented in the different HF including training of health workers, donation of IPC kits, supportive supervision during the implementation of IPC activities, and monetary reward. IPC practices in HF were assessment every two weeks during the intervention period to measure the impact. The IPC assessment tool had 34 questions aggregated in 8 different thematic areas: triage and isolation capacity, IPC committee in HF, hand hygiene, PPE, decontamination and sterilization, linen management, hospital environment and Waste management. Data were analysed using descriptive statistics and analytical approaches according to assumptions. R software (version 4.0.3) was used for all the analyses and a p-value of 0.05 was considered as the threshold for statistically significant results.

**Results:**

Among 69 HF involved in this study, 48 were private facilities and 21 state facilities. The median baseline IPC score was 44% (IQR: 21–65%); this IPC median score reached respectively after 2, 4, 6 and 8 weeks 68% (IQR: 59–76%), 79% (71–84%), 76% (68–85%) and 79% (74–85%). The improvement of IPC score was statistically significative. Spearman’s rank-order correlation revealed the associated between proportion of trained HW and IPC score performance after 8 weeks of interventions (rs = .280, p-value = 0.02).

**Conclusion:**

Pay for Performance Strategy was proved effective in improving healthcare facilities capacity in infection prevention and control practice in context of 2018 EVD outbreak in Nord Kivu. However, the strategy for long-term sustainability of IPC needs further provision. More studies are warranted on the HW and patients’ perceptions toward IPC program implementation in context of Nord Kivu Province.

## Introduction

Lessons learned from previous Ebola outbreaks showed that health workers (HW) are at highest risk of exposure to Ebola virus disease (EVD) [1–6]. During the 2018–2020 EVD outbreak in the Democratic Republic of Congo (DRC), a total of 3,481 Ebola cases were reported among which 160 (27.6%) were HW and 2,299 (66%) deaths, making this the second-largest documented Ebola outbreak after the 2014–2016 epidemic in West Africa, which resulted in 28,600 cases and 11,325 deaths [2, 7]. A number of IPC strategies have been developed in Sierra Leone to strengthen health systems in order to keep the health facilities (HF) prepared against EVD outbreaks [3].

To strengthen the DRC’s health system in IPC, a comprehensive ring approach was advocated by several IPC experts based on other EVD outbreak’s lessons learned [4–6, 8]. Barriers that play a role in the implementation of IPC interventions have been identified, and they are likely associated with poor HF IPC performance [8]. Additional IPC challenges were reported in Western Africa outbreak [9]. A study by Shoman et al. revealed that shortage in diverse key aspects (health workforce, information and research, medical products and technologies, financing, and leadership and governance) had a negative effect on the EVD outbreak mitigation [10]. WHO states that “a well-functioning health system working in harmony is built on having trained and motivated health workers, a well-maintained infrastructure, and a reliable supply of medicines and technologies, backed by adequate funding, strong health plans and evidence-based policies” [10]. The payment approach known as “pay-for-performance strategy (PPS)” has been widely adopted with the aim of improving the quality of healthcare. Nonetheless, less is known about the PPS approach to effectively improve the IPC practices in both private and public health facilities in the context of an EVD outbreak. Some studies have shown that PPS may motivate hospitals to improve the quality of service delivery [11–17]. 

PPS provides financial incentives to HW or HF based on the achievement of pre-specified performance targets. It has been widely conducted in health systems across low and middle-income countries (LMICs) where their impact was evaluated to improve hospital service delivery [18]. Although many challenges related to the implementation of IPC measures were always identified during EVD outbreaks (including lack of medical suppliers in various health facilities and procurement systems), data on the impact of PPS in improving IPC practices in DRC is still deficient.

This study assesses the impact of PPS in improving HF capacity in IPC practices in the context of 20,218–2020 DRC EVD outbreak.

## Methods and materials

### Study design, settings and participants

We have conducted a prospective analytical quasi-experimental study in Beni City (North-Kivu, east of DRC), one of the EVD outbreak epicentre, to assess the impact of a pay for performance strategy on the improvement of IPC performances in HF during the Ebola outbreak from December 2018 to February 2019.

We included HF that have admitted at least 1 EVD case, HF with admission services, HF in districts with at least 3 EVD confirmed cases. We numbered and recorded the HW and beds of each HF. HF were categorized according to the number of beds they had: HF category 1 had more than 39 beds, HF category 2 had 20–39 beds, HF category 3 had 5–19 beds, and HF category 4 had 4 beds maximum.

### Interventions

Interventions included training of HW, donation of IPC/WASH kits, supportive supervision during the implementation of IPC activities, and monetary reward.

A piloting committee oversaw the overall implementation of the PPS. It comprised seven IPC experts and one program manager. We established a health area prevention committee in each health area comprising selected HF. Each prevention committee was formed by the registered nurse, the representative private HF, the representative of the healers, and the neighbourhood chief. Prevention committee’s members provided technical support to focal points in HF and IPC mentors in the rollout of activities and the piloting committee in the implementation of the project. They weekly organized, activities’ meeting.

The heads of each HF selected signed a contract of performance in which they acknowledged supporting IPC activities versus a financial incentive allocated to the facility as a reward of the IPC performance. We recruited 24 IPC mentors (one IPC mentor for three HF) to play following roles: assessment of IPC performance in HF, identification of IPC gaps, suggestion of key actions for improvement, provide assistance to HF in the planning of IPC activities and training of HW.

To reinforce IPC measures in HF, the following items constituting IPC/WASH kits were donated: examination gloves, thermoflash, N95 masks, surgical masks, rubbish bins, alcohol-based hand rub gel, paper towels, sharp containers, dishwashing gloves, stickers with Ebola toll-free number, soap, raincoats, face shields, chlorine, laboratory coat, aprons, goggles, different posters (EVD case definition, hand hygiene and waste management), gumboots, plastic chair, plastic table, hand washing tap buckets and small waste disposal incinerators. An IPC focal point was appointed among HW in the concerned facility.

A three-day training session was conducted as per WHO and DRC’s Ministry of Health modules. The first day of the training focused on basic principles of EVD and IPC standard precautions, triage and patients’ isolation, and hospital-based surveillance. Day 2 training addressed injection safety, cleaning and decontamination of the environment, waste management, safe and dignified burial, and psychological aspects of EVD. Day 3 training supported IPC ring strategy, IPC kit constitution, assessment of IPC in HF and stock management.

### Outcome and monetary incentives

IPC practices in HF were assessment every two weeks. The IPC assessment tool comprised 34 questions aggregated in 8 sections: triage and isolation capacity, IPC committee in HF, hand hygiene, PPE, decontamination and sterilization, linen management, hospital environment and Waste management.

Depending on the category of the HF and the IPC score generated, an amount of money was allocated to that facility. HF with IPC scores > 80% would get 100% of the total amount, HF with an IPC scores between 60 and 80% perceived 80%, HF with 50–59% scores received 60%, and HF with scores < 50% were not rewarded.

### Data management and analysis

We used an IPC assessment tool in Microsoft Excel^®^ to manage IPC indicators. Data were summarized in descriptive analysis. Category variables were presented as frequencies and percentages; median and interquartile range (IQR) were used to summarize numerical data as they presented an asymmetric distribution using the Shapiro Wilk test. To compare IPC scores before and after the implementation of the intervention, we use the Friedman test (nonparametric test that compares medians of two paired groups). We performed the Man-Whitney U and Kruskal–Wallis H tests to compare variable with asymmetric distribution for 2 and more than 2 unpaired groups, respectively. Spearman’s rank-order correlation was run to determine the relationship between the proportion of HWs trained and the IPC score after 8 weeks of intervention. P-value of 0.05 was considered statistically significant for all analyses. The R software (version 4.0.3) was used for these statistics analyses.

## Results

We included 69 HF coming from 15 health areas in the district of Beni among which 48 were private and 21 public. Eleven (15.9%) HF were listed in the category 1, 21 (30.4%) HF in the category 2, 32 (46.3%) in the categories 3 and 5 (7.2%) in the category 4. The median number of beds in HF was 18 (IQR: 8–27). We recorded 1,121 HW from 69 HF with a median number of 12 HW per HF (IQR: 6–18 HW). Five hundred sixty-four HW were trained in IPC practices (50.3% of all the HW) and 874 HW (77.9%) were immunized with rVSV-ZEBOV-GP vaccine (Merck, Ltd) (see Table 1).

**Table 1:** General characteristics of the health facilities & health workers

	Category 1	Category 2	Category 3	Category 4	Total
HF					
Public HF	6	8	7	0	21
Private HF	5	13	25	5	48
N° of HW	437	367	286	31	1121
N° of HW trained	235 (53.7%)	190 (51.7%)	135 (47.2%)	4 (12.9%)	564 (50.3)
N° of HW vaccinated	367 (83.9%)	297 (80.9%)	190 (47.2%)	20 (64.5%)	874 (77.9%)

N°: Number, HF: Health Facilities, HW: Health Workers, Category 1: HF with less than 5 beds, Category 2: HF with 5–19 beds, Category 3: HF with 20–39 beds, Category 4: HF with more than 39 beds

Seventy percent of the money allocated to HF was used to buy IPC supplies and all the HF workers shared the remaining 30% as an incentive bonus. Table 2 gives a summary of the percentage of amount allocated to HF according to their performance and the category they belonged to.

**Table 2:** Monetary incentives to HF according to the IPC scores and HF categories

IPC score	% monetary incentive	Cat 1 (USD)	Cat 2 (USD)	Cat 3 (USD)	Cat 4 (USD)
> 80%	100%	1633	858	513	125
60–79%	80%	1307	687	410	100
50–59%	60%	980	515	308	75
< 50%	0%	0	0	0	0

HF: Healthcare Facility; Cat 1, 2, 3, 4: Healthcare Facility Category 1, 2, 3, 4, respectively HF with less than 5 beds, 5–19 beds, 20–39 beds and more than 40 beds; IPC: infection prevention and control

Spearman’s rank-order correlation test was run to determine the relationship between the proportion of HWs trained and the IPC score after 8 weeks of intervention. There was a slight positive correlation between these two variables, which was statistically significant (rs = 0.280, p-value = 0.02) (Table 3).

**Table 3:** Correlation between the proportion of HW trained per facility and the IPC score after 8 weeks of intervention

Correlations				
			% HW trained	IPC score week 8
Spearman’s rho	% HW trained	Correlation Coefficient	1.000	0.280**
		Sig (2 tailed)		0.02
		N	69	69
	IPC score week 8	Correlation Coefficient	0.280**	1.000
		Sig (2 tailed)	0.02	
		N	69	69

**Correlation is significant at the 0.05 level (2-tailed)

**Fig. 1:**
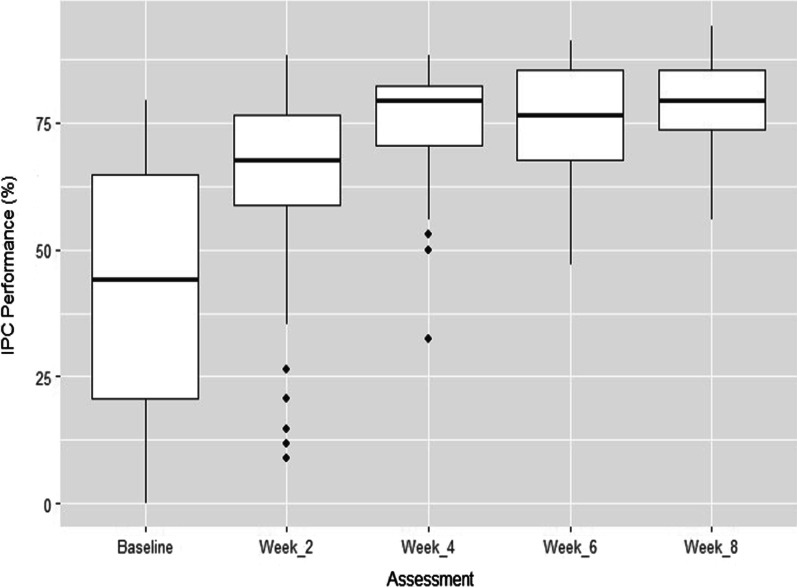
IPC performance by improvement from the baseline to the final assessment. Baseline: baseline IPC score, Week_2: IPC score after 2 weeks of intervention, Week_4: IPC score after 4 weeks of intervention, Week_6: IPC score after 6 weeks of intervention, Week_8: IPC score after 8 weeks of intervention

The median baseline IPC score was 44% (IQR: 21–65%) and it has progressively improved to 68% (IQR: 59–76%), 79% (71–84%), 76% (68–85%) and 79% (74–85%) (p-value < 0.001) after 2, 4, 6, and 8 weeks, respectively (Fig. 1).

The overall analysis of IPC performance by HF categories shows that bigger facilities in terms of the number of beds tend to have better IPC performances (Table 4). For baseline assessment, HF from category 1 had a median IPC score of 55.8% as the median IPC score of those of category 4 was 17.6% (p-value: 0.002). The follow-up assessment also displayed similar figures in terms of the difference in performance between different categories of HF.

**Table 4:** IPC performance by healthcare facility’s category

IPC score	Cat 1 (%)	Cat 2(%)	Cat 3(%)	Cat 4(%)	p-value
Baseline	55.8	52.9	33.8	17.6	0.002
After 2 weeks	76.4	73.5	61.7	47.0	< 0.001
After 4 weeks	79.4	79.4	75.0	55.8	0.041
After 6 weeks	82.3	85.2	73.5	67.6	0.016
After 8 weeks	82.3	85.2	76.4	79.4	0.002

The results show that there was a significant improvement in IPC performances for triage capacity, IPC program at HF, multimodal strategy for hand hygiene, PPE use, waste management, hospital sanitation and linen management after 8 weeks of IPC package implementation among the selected HF (p < 0.001). However, there was no improvement in practice for sterilization of medical equipment and decontamination of surfaces score IPC (p**-**value = 0.3) (Table 5).

**Table 5:** Compare median IPC score between baseline and after intervention based on 8 IPC key performance indicators

IPC thematic area	Baseline	Week 2	Week 4	Week 6	Week 8	p-value
Triage	60	80	80	80	80	< 0.001
IPC program	50	100	100	100	100	< 0.001
Hand hygiene	60	80	80	80	80	< 0.001
PPE use	25	50	100	75	75	< 0.001
Sterilization and decontamination	66	66	66	66	66	0.3
Linen management	33	33	33	66	100	< 0.001
Hospital environment	25	50	75	75	75	< 0.001
Waste management	50	83	83	83	83	< 0.001

IPC: infection prevention and control; PPE: personal protective equipment; Baseline: baseline IPC score, Week 2: IPC score after 2 weeks of intervention, Week 4: IPC score after 4 weeks of intervention, Week 6: IPC score after 6 weeks of intervention, Week 8: IPC score after 8 weeks of intervention

## Discussion

This study, conducted in North Kivu during the 2018 EVD outbreak, was the first of its kind in DRC assessing the impact of PPS in improving IPC practices. Results showed that there was a statistically significant change between mean IPC performance score at baseline and after 2, 4, 6 and 8 weeks.

There was a moderate positive correlation between the proportion of HWs trained in HF and the IPC scores after week 8 of implementation of the PPS strategy. A number of key IPC performance indicators were improved from the baseline to week 8 of intervention. These included triage functionality, implementation of IPC committee and program at facility level, multimodal strategy for hand hygiene, PPE use, waste management, hospital sanitation and linen management. The implementation of improvement plan for each IPC component consisted not only in providing monetary incentive but as well by training HW, supplying equipment and materials needed for IPC practices and supportive supervision at the point of care. Multimodal strategies have shown its efficiency in improving IPC practices [19, 20]. A systematic review summarizing 57 studies has highlighted the role of multimodal strategies in the improvement of hand hygiene compliance. Of the interventions described in that study, education and training were the most common. Other interventions were performance feedback, hands hygiene reminders, and provision of hands hygiene materials and/or infrastructure including alcohol-based hands rub [21].

In our study, however, there was no improvement in IPC performance for sterilization of medical equipment and decontamination of surfaces. This can be explained by the fact that sterilization involves the use of specialized materials that most of the HF did not have before the outbreak and during the response phase, these materials were distributed in a very low quantity.

This study also displayed that smaller HF were less compliant to IPC measures compared to bigger HF. In the Beni District, many of these small private HF did not have required infrastructure and materials, and HW did not, in most of the cases, required qualification for medical practice. Moreover, due to a high number of HW, bigger facilities were able to identify an IPC focal point fully dedicated to IPC work; but in small facilities, the person identified for IPC activities was mostly multitasking.

Our findings support many studies evaluating the impact of PPS to improve IPC practice without negative effects on the health system if the resilience strategies are implemented [15–18, 22–24]. A study conducted on the effects of PPS in 260 hospitals compared to 780 hospitals without PPS (control) showed that more than half of PPS hospitals achieved high performance scores, compared to less than a third of control hospitals. However, the scores of the two groups were identical overtime [23]. Our findings support that tailoring pay-for-performance programs to strengthen health systems could have the greatest effect on the quality of healthcare provided in low-income settings such as DRC. PPS was found to be an effective approach to motivate HW to increase the quality of care they are providing in their HF, by increasing the rate of adherence to IPC measures and other health interventions in many outbreaks in low-income countries.

During the 2014–2016 EVD outbreak, affected countries shared similar health system weaknesses as highlighted during the 2018–2020 EVD outbreak in DRC. These may include insufficient surveillance systems and lack of standardized IPC programs implemented in local HF. This resulted in an increase in the incidence of nosocomial EVD infection as documented during outbreaks in Sierra Leone, Guinea and DRC [22–24]. Despite tremendous efforts performed and important financial resources mobilized during the 2018–2020 EVD outbreak in DRC, some challenges remained uncovered due to the lack of long-term sustainability. Training of HW in HF improved significantly IPC performance, helping to minimize nosocomial transmission. The association between IPC training of HW and the improvement of IPC performance was reported in HF in a study conducted in one municipality of Conakry/Guinea during the 2014 EVD outbreak [25]. Moreover, IPC cascade training using PPS approaches compared to cascade training without PPS approaches is likely to result in higher IPC performance score, regardless of the type of HF and HW. Although our results are supported by the study conducted in Guinea which involved different partners during the outbreak, more qualitative and quantitative studies should be conducted to assess the impact of PPS on the long-term sustainability in low- and middle-income countries.

### Limitations

Lack of control group is likely associated with limited external validity of the study results. HF included in this study may not be representative of all HF of the province of North-Kivu. This was due to funding limitation and inaccessibility of some areas where army groups are established. The IPC assessment tool did not include quality assurance on hospital management from different departments of HF [26]. As result, data on the functionality of HF beyond IPC were not assessed. Other approaches should be designed, with more effective methods of training and cognitive interventions. Finally, HW perceptions and patients’ appreciations were not qualitatively evaluated as part of implementation process of this strategy as well as cost-effectiveness analysis were not conducted to mobilize additional human resources and funds as part of participatory research which may include policy-makers and other stakeholders.

## Conclusion

Pay for Performance Strategy was proved effective in improving health facilities capacity in IPC practices in context of 2018 EVD outbreak in Nord Kivu associated with the IPC multimodal strategies which include continuing HW training, supportive supervision, donation of IPC/WASH kits, facility quality improvement plans, and allocated the national budget to strengthen IPC programs. Additional studies are warranted on HW and patients’ perceptions toward IPC program implementation considering limited accessibility and security context of the region.

## Data Availability

The data that support the findings of this study are available from the corresponding author, LK, upon reasonable request.

## Abbreviations

IPC: Infection prevention and control
EVD: Ebola virus disease
DRC: Democratic Republic of the Congo
HW: Health workers
HF: Health facilities
PPS: Pay for performance strategy
